# Protein phosphorylation associated with drought priming-enhanced heat tolerance in a temperate grass species

**DOI:** 10.1038/s41438-020-00440-8

**Published:** 2020-12-01

**Authors:** Xiaxiang Zhang, Lili Zhuang, Yu Liu, Zhimin Yang, Bingru Huang

**Affiliations:** 1 College of Agro-grassland Science, Nanjing Agricultural University, 210095 Nanjing, China; 2 Department of Plant Biology, Rutgers University, New Brunswick, NJ 08901 USA

**Keywords:** Abiotic, Protein-protein interaction networks

## Abstract

Protein phosphorylation is known to play crucial roles in plant tolerance to individual stresses, but how protein phosphorylation is associated with cross-stress tolerance, particularly drought priming-enhanced heat tolerance is largely unknown. The objectives of the present study were to identify phosphorylated proteins and phosphorylation sites that were responsive to drought priming and to determine whether drought priming-enhanced heat tolerance in temperate grass species involves changes in protein phosphorylation. Comparative analysis of phosphoproteomic profiles was performed on leaves of tall fescue (*Festuca arundinacea*) exposed to heat stress (38/33 °C, day/night) with or without drought priming. A total of 569 differentially regulated phosphoproteins (DRPs) with 1098 phosphorylation sites were identified in response to drought priming or heat stress individually or sequentially. Most DRPs were nuclear-localized and cytosolic proteins. Motif analysis detected [GS], [DSD], and [S..E] as major phosphorylation sites in casein kinase-II and mitogen-activated protein kinases regulated by drought priming and heat stress. Functional annotation and gene ontology analysis demonstrated that DRPs in response to drought priming and in drought-primed plants subsequently exposed to heat stress were mostly enriched in four major biological processes, including RNA splicing, transcription control, stress protection/defense, and stress perception/signaling. These results suggest the involvement of post-translational regulation of the aforementioned biological processes and signaling pathways in drought priming memory and cross-tolerance with heat stress in a temperate grass species.

## Introduction

Plants growing in natural environments are exposed to multiple abiotic stresses which may occur sequentially or simultaneously^1^. Plants previously exposed to a single stress factor or primed by pre-exposure to stress may acquire mechanisms that enable tolerance to subsequent stresses, which is referred to as stress priming^2,3^. Drought priming has been reported to improve plant resistance to subsequent stresses, including heat, chilling, and drought stresses in various plant species^4–6^. However, the mechanisms by which drought priming enhances plant heat tolerance are not yet well understood, although great efforts have been taken in this research area.

Enhanced tolerance by drought priming has been related to the maintenance in photosynthesis, activation of antioxidant defense systems and accumulation of compatible solutes^5–7^. Lipidomic analysis found that lipid reprogramming was involved in drought priming-induced heat tolerance in tall fescue (*Festuca arundinacea*)^8^. Using a time-course comparative transcriptome analysis, Harb et al^7^. presented that a moderate drought stress triggered accumulation of abscisic acid (ABA) and induction of associated signaling genes, resulting in an early avoidance response to later drought stress in *Arabidopsis thaliana*. Proteomic analysis has found a large number of proteins, such as fructose-1,6-bisphosphatase involved in sucrose synthesis, glutathione-S-transferase and catalase involved in antioxidation, chaperonin, and heat shock proteins were up-regulated by drought priming^6,9^. Previous researches have shown that rapid accumulation of heat shock proteins is critically important for drought- and heat-priming of plants^9,10^. In addition to modification at gene transcript and protein levels, drought priming may affect protein post-translational modifications (PTMs) which affect the structure, localization, and activity of proteins, ultimately regulating plant growth and development, and mediating plant responses to environmental stimulus^11,12^. Protein phosphorylation in the process of PTMs plays crucial roles in plant stress tolerance by transmitting intracellular signals from the cell surface to the nucleus to regulate cellular functions of proteins^13^. However, how drought-priming regulation of protein phosphorylation is related to acquired heat tolerance is not well understood.

Profiling phosphoproteomic changes, such as specific phosphoproteins and phosphosites, can help understand cellular regulatory mechanisms of stress defense^14^. For example, large-scale quantitative phosphoproteome analysis in tomato (*Solanum lycopersicum*) plants identified novel signaling cascades involving different kinases that regulate cold acclimation^15^. Several studies reported phosphoproteins responsive to drought or heat stress individually^12,16^. For example, Liu et al.^12^ identified up-regulated phosphoproteins of SR-rich splicing factors and heat shock proteins involved in heat stress responses in grape (*Vitis vinifera*) leaves. Yuan et al.^16^ found that significant changes in phosphorylation levels of proteins involved in signal transduction and material transmembrane transport were associated with plant response to drought stress in *Brachypodium distachyon* seedlings. To our knowledge, little is known about how protein phosphorylation in terms of phosphoproteins and phosphorylation sites may be involved in cross-stress tolerance, particularly drought priming-enhanced thermotolerance.

We hypothesized that protein phosphorylation may be involved in plant stress memory, unique phosphopeptides for stress protection or defense may be induced or enriched by drought priming and may contribute to plant tolerance to subsequent heat stress. Our previous study has shown that drought priming-enhanced heat tolerance was characterized with lipid reprogramming in tall fescue which is one of the most widely used turfgrass species in the world^8^. The main objective of the present study was to identify phosphorylated proteins and phosphorylation sites responded to drought priming and to determine whether drought priming-enhanced heat tolerance involves protein phosphorylation in tall fescue. These objectives will be addressed by comparative analysis of phosphoprotein profiles between drought-primed to non-primed plants subsequently exposed to heat stress. The information will gain insights into the roles of protein phosphorylation in cross-stress tolerance in temperate plant species.

## Results

### Phosphoproteins identified in tall fescue leaves

A total of 1181 phosphopeptides with 3901 phosphosites were identified in all samples. Among all, 753 differentially regulated phosphopeptides (fold-change ratio > 1.3, *p* < 0.05) corresponded to 569 proteins (differentially regulated phosphoproteins, DRPs) with 1098 phosphorylation sites in response to drought priming, heat stress, and heat stress with drought priming were identified (Fig. S1, Supplemental Table S1). The majority of the phosphopeptides was single phosphorylated (455, 60.42%), while 251 (33.33%) and 47 (6.24%) peptides carried two and three phosphorylation modifications, respectively (Fig. S1). The most prominent phosphorylated amino acid detected was pSerine (92.44%) (Fig. S1).

### Differentially regulated phosphopeptides in response to individual or sequential drought priming and heat stress

**Fig. 1:**
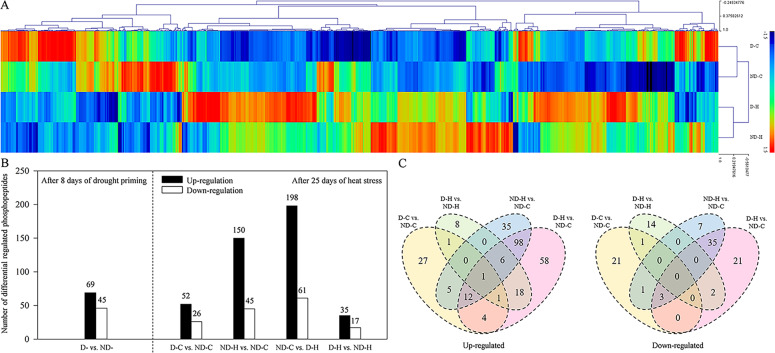
Statistical analysis of phosphoproteomics of tall fescue plants under different treatments. **a** Hierarchical clustering of all differently regulated phosphopeptides among different treatments (*p*-value < 0.05 and fold-change > 1.3, data were calculated by z-score normalization). **b** Number of up- and down regulated phosphopeptides in tall fescue leaves in response to drought priming and heat stress. **c** Venn diagram of significantly changed phosphoproteins distributed between different treatments. ND-, no drought priming; D-, drought priming. ND-C, non-drought priming + control temperature; D-C, drought priming + control temperature; ND-H, non-drought priming + subsequent heat stress; D-H, drought priming + subsequent heat stress

The heat map (Fig. 1a) showed that a number of phosphopeptides were differentially regulated in drought-primed plants (D-H) from those in non-drought primed plants (ND-H) under heat stress. The differentially regulated phosphopeptides were placed in four clusters. The first cluster included phosphopeptides primarily showing decreases in the abundance level under heat stress; the second and third clusters included phosphopeptides that showed increases in the abundance level under D-H and ND-H treatments, respectively; the fourth cluster showed phosphopeptides primarily increased in the abundance level by drought priming (D-C or D-H). A total of 52 phosphopeptides were up-regulated while 26 were down-regulated due to drought priming (D- vs. ND-) (Fig. 1b, Table S2). A total of 150 phosphopeptides were up-regulated and 45 were down-regulated in response to heat stress without drought priming (ND-H vs. ND-C). With drought priming, 35 phosphopeptides were up-regulated and 17 were down-regulated under heat stress (D-H vs. ND-H) (Fig. 1b, Table S3). A total of 301 phosphopeptides showed significant differences in abundance levels for drought-primed plants between heat stress (D-H) and non-stress control (D-C), with 222 up-regulated and 79 down-regulated. A total of 35 phosphopeptides were up-regulated and 17 down-regulated in response to heat stress in drought-primed (D-H) compared with non-primed plants (ND-H) (Fig. 1b and Fig. 1c).

**Fig. 2:**
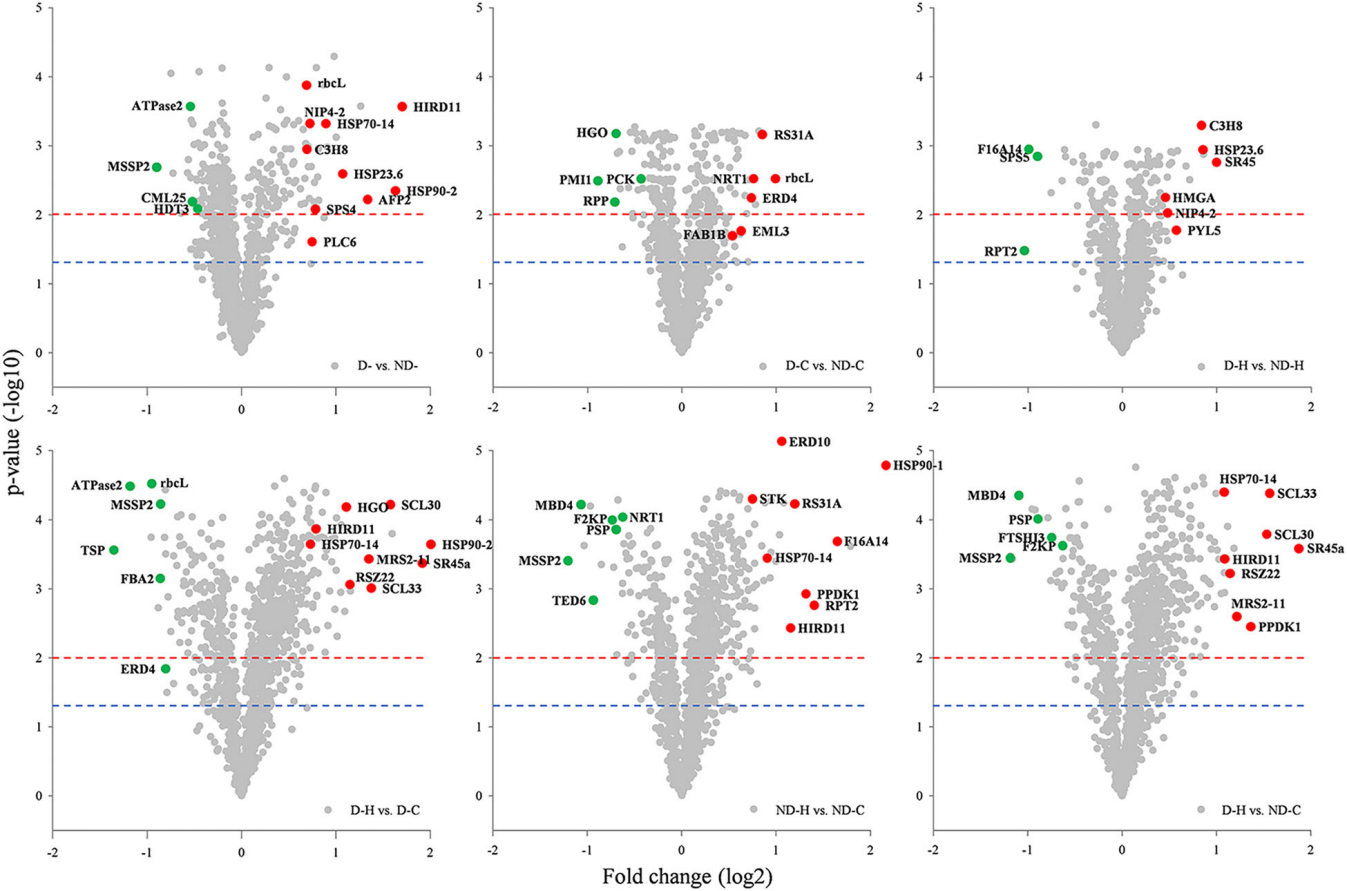
Volcano plots of differentially regulated phosphopeptides of tall fescue between different treatments. Note: Green and red filled points represent significantly downregulated and upregulated phosphopeptides, respectively (*p* < 0.01). Blue and red dash line indicated the confidence threshold of *p* < 0.05 and 0.01, respectively. ND-, no drought priming; D-, drought priming. ND-C, non-drought priming + control temperature; D-C, drought priming + control temperature; ND-H, non-drought priming + subsequent heat stress; D-H, drought priming + subsequent heat stress

To better demonstrate changes in phosphorylation levels for specific phosphopeptides in responses to drought priming and heat stress, volcano plots exhibiting upregulation or down-regulation in different treatments were constructed (Fig. 2). The significantly regulated phosphopeptides which had low p-value and high fold-change were displayed at the top left and right of the volcano plots. Drought priming up-regulated the phosphorylation level of dehydrin HIRD11, aquaporin NIP4-2, zinc-finger CCCH domain-containing protein 8 (C3H8), phosphoinositide phospholipase C 6 (PI-PLC6), heat shock protein 23.6 (HSP23.6) and HSP90-2, while it down-regulated ATPase 2, mnosaccharide-sensing protein 2 (MSSP2) and calmodulin-like protein 25 (CML25). Heat stress induced the phosphorylation of phosphate dikinase 1 (PPDK1), HSP90-1, and HSP90-2, with the highest increase in their abundance level up to 4-8 fold. Under heat stress, drought primed plants had higher phosphorylation level of HSP23.6, C3H8, NIP4-2, serine/arginine-rich splicing factor (SR45), high mobility group A protein (HMGA), HMGB2 and abscisic acid receptor PYL5, compared to non-primed plants (Fig. 2).

### Functional annotation and subcellular localization of differentially regulated phosphoproteins

The up-regulated phosphoproteins by drought priming were classified into 17 functional categories, including “RNA related” (11 proteins, 15.94%), “stress related” (10 proteins, 14.49%), “protein related” (6 proteins, 8.70%), “signaling related” (5 proteins. 7.25%), and “transport related” (4 proteins, 5.80%) (Fig. S2, Table S2). According to their subcellular locations, most of those phosphoproteins identified were nuclear-localized proteins (25 proteins, 36.23%), followed by cytosolic proteins (20 proteins, 28.99%), plasma membrane proteins (11 proteins, 15.94%), and plastidial proteins (11 proteins, 15.94%). The down-regulated phosphoproteins by drought priming were classified into 11 functional categories, including “RNA related” (12 proteins, 26.09%), “transport related” (7 proteins, 15.22%), “protein related” (4 proteins, 8.70%), and “signaling related” (3 proteins. 6.52%). Those phosphoproteins were identified as mostly nuclear-localized proteins (20 proteins, 43.48%), plasma membrane proteins (8 proteins, 17.39%), cytosolic proteins (7 proteins, 15.22%) and vacuole proteins (4 proteins, 8.70%).

Comparing to ND-H, D-H treated plants had up-regulated phosphorylation level of proteins involving in “RNA related” (14 proteins), “stress related” (3 proteins), “transport related” (2 proteins) and “hormone metabolism” (2 proteins), while had down-regulated phosphorylation level of proteins involving in “RNA related” (5 proteins), “signaling related” (3 proteins), “stress related” (2 proteins) and “protein related” (2 proteins). Those DRPs were mostly localized in nuclear and cytosol, with 23 and 7 proteins up-regulated, 8 and 4 proteins down-regulated, respectively.

### Gene ontology analysis of differentially regulated phosphoproteins

**Fig. 3:**
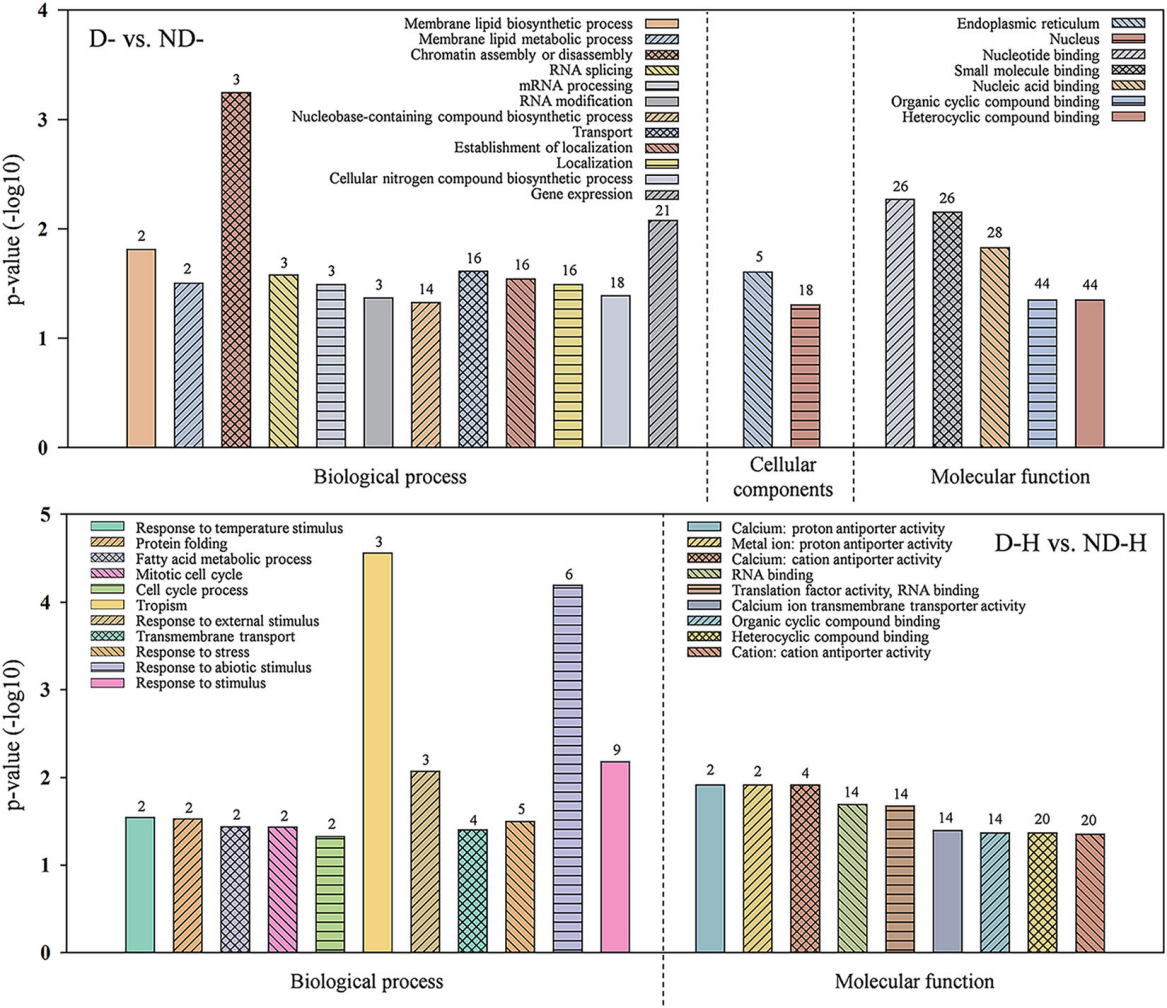
Enrichment analysis based on GO terms of differentially expressed phosphoproteins in response to drought priming and subsequent heat stress. ND-, no drought priming; D-, drought priming. ND-H, non-drought priming + subsequent heat stress; D-H, drought priming + subsequent heat stress

Gene ontology (GO) category enrichment analysis indicated that DRPs responsive to drought priming (D- vs. ND-) were enriched in various biological processes, cellular components, and molecular functions (Fig. 3). Phosphoproteins were enriched in GO categories related to “membrane lipid biosynthetic process”, “RNA splicing”, “chromatin assembly or disassembly”, “localization”, “transport” and “gene expression” were enriched in the “biological process”. Endoplasmic reticulum and nucleus were enriched in the “cellular component”. Regarding the “molecular function”, DRPs were preferentially cataloged into “binding”, including “nucleotide binding”, “small molecule binding”, “nucleic acid binding”, “organic cyclic compound binding” and “heterocyclic compound binding”. In response to heat stress, 52 DRPs in drought-primed plants (D-H vs. ND-H) were enriched in biological processes and molecular functions. Among them, 9 DRPs were enriched in the biological process of “response to stimulus” and others were associated with biological processes of “fatty acid metabolic process”, “transmembrane transport” and “protein folding”, while in molecular function, “heterocyclic compound binding” (20 proteins) and “cation: cation antiporter activity” (20 proteins) were the most highly represented group. All enriched GO terms of these two comparisons were shown in Table S5.

### Phosphorylation motif analysis

**Fig. 4:**
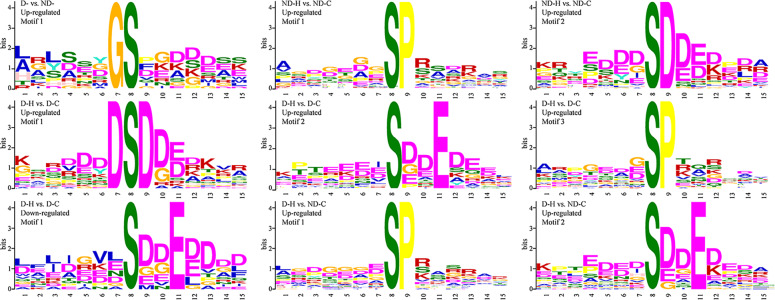
Phosphorylation motifs enriched from peptides with different modification levels in response to drought priming and subsequent heat stress in tall fescue. The phosphorylation motifs were identified using the Motif‐X algorithm. ND-, no drought priming; D-, drought priming. ND-C, non-drought priming + control temperature; D-C, drought priming + control temperature; ND-H, non-drought priming + subsequent heat stress; D-H, drought priming + subsequent heat stress

To further evaluate sequence conservation at phosphosites and for predicting the associated kinases, sequences of significantly changed phosphopeptides between different treatments were submitted for phosphorylation motif analysis (Fig. 4, Table S4). [GS] motif was enriched in up-regulated phosphopeptides by drought priming (D- vs. ND-). Two motifs ([SP] and [S..E]) were enriched in up-regulated phosphopeptides due to heat stress (ND-H vs. ND-C). Three phosphorylation motifs, [DSD], [S..E], and [SP] were enriched in up-regulated phosphopeptides in drought-primed plants under heat stress (D-H vs. D-C).

### Protein–protein interaction (PPI) analysis

**Fig. 5:**
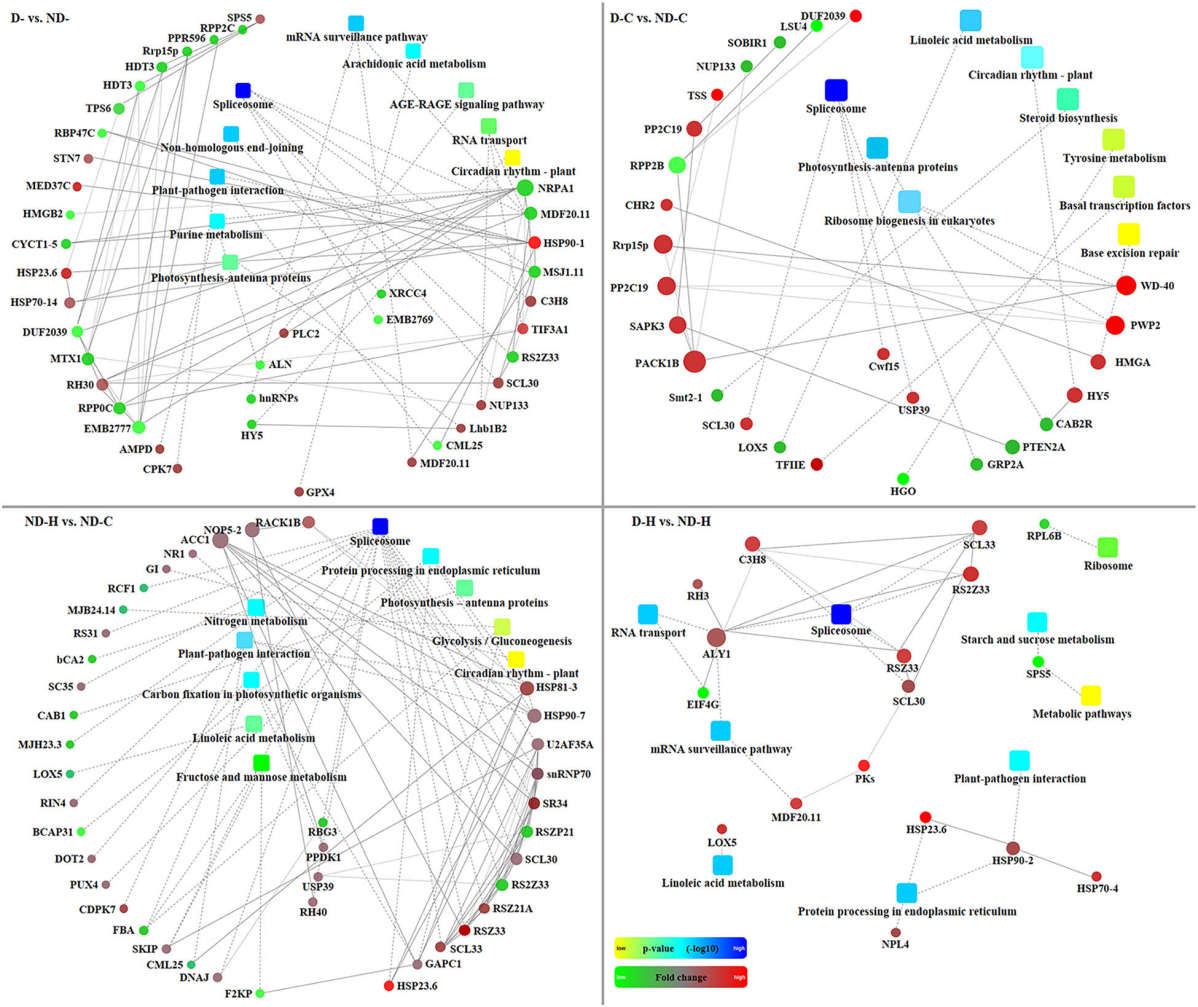
Protein–protein interaction network among differentially regulated phosphoproteins in response to drought priming and subsequent heat stress. Network mapping were visualized by OmicsBean based on fold change of protein and *p*-value of biological process enrichment. Proteins are indicated by circle nodes with gradient color (red, up-regulation; green, down-regulation). The names of the differentially regulated phosphoproteins were presented by the names or the locus numbers of the homologous proteins in Arabidopsis. The sizes of nodes represented the number of interactions. Squares indicate biological processes, which were colored with a yellow to blue gradient (smaller to larger *p*-value, respectively). ND-, no drought priming; D-, drought priming; ND-C, non-drought priming + control temperature; D-C, drought priming + control temperature; ND-H, non-drought priming + subsequent heat stress; D-H, drought priming + subsequent heat stress

PPI networks for DRPs responsive to drought priming or heat stress with drought priming were analyzed based software for protein interaction prediction (OmicsBean, http://www.omicsbean.cn/) and visualized using Cytoscape (version 3.5, Fig. 5). The PPI network affected by drought priming (D- vs. ND-) was composed of 58 DRPs and the largest subnet was “spliceosome” including 4 down-regulated proteins (MDF20.11, RS2Z33, MSJ1.11, EMB2769) and 2 up-regulated proteins (C3H8 and SCL30). The other subnets of PPI affected by drought priming were associated with “RNA transport”, “mRNA surveillance pathway”, “plant-pathogen interaction” and “photosynthesis”. PPI networks affected by heat stress (ND-H vs. ND-C) included proteins involved in “spliceosome”, “protein processing in endoplasmic reticulum”, “fructose and mannose metabolism” and “carbon fixation in photosynthetic organisms”. Heat stress with drought priming (D-H vs. ND-H) resulted in the alteration of 18 proteins involved in PPI. Six proteins (C3H8, ALY1, RSZ33, RS2Z33, SCL33, and SCL30) were in “spliceosome”. HSP23.6 and HSP70-4 were both up-regulated and participated in “plant-pathogen interaction” and “protein processing”. LOX5 was up-regulated and participated in “linoleic acid metabolism” while SPS5 was down-regulated and participated in “starch and sucrose metabolism”.

## Discussion

Protein phosphorylation and dephosphorylation status affect the structure, activity, subcellular distribution of proteins, influence the interaction with other protein, and thereby modulate plant growth and stress responses^17^. This study first identified a total of 753 phosphorylated peptides induced by drought priming or heat stress individually and heat stress with drought priming in a cool-season grass species. Among the large number of DRPs, specific DRPs up-regulated by drought priming and sustained during the later heat stress were particularly interesting, which could be related to priming-enhanced stress tolerance. Those DRPs mainly classified into four functional categories, including RNA splicing, transcription control, stress protection, and stress perception/signaling are discussed in detail below.

### Upregulation of phosphorylated proteins in RNA splicing associated with drought priming and priming-induced heat tolerance

RNA splicing is the process transforming pre-mRNAs to mature mRNAs, which is critical for proper translation of mRNA into a protein^18,19^. Serine/arginine-rich proteins are a conserved family of RNA-binding proteins which are involved in spliceosome assembly and alternative pre‐mRNA splicing, and are key players in plant development and response to abiotic stresses^18^. Previous studies have shown that phosphorylation level of serine/arginine-rich splicing factors was activated under drought stress^20^. Our results demonstrated that the phosphorylation level of four serine/arginine-rich splicing factor was up-regulated by drought priming. In addition, recent studies proved the role for alternative pre-mRNA splicing as a plant ‘molecular thermometer’, expanding proteomic diversity and allowing plants to quickly adjust gene expression at the post-transcriptional level^19^. In our study, the phosphorylation levels of 10 proteins involved in “RNA splicing”, including SR45, RS2Z33, SCL33, and SCL30, were significantly up-regulated in D-H compared with ND-H, suggesting that the enhanced phosphorylation level of splicing factors could be induced by drought priming, which could facilitate enhanced heat tolerance.

### Phosphorylated proteins in transcriptional control associated with drought priming and priming-induced heat tolerance

Transcriptional control is a major mechanism for regulating gene expression in a cell or organism^21,22^. It is known that exposure of plants to unfavorable environmental conditions can activate both transient and stable transcriptional changes, and recently chromatin-mediated transcriptional regulation has been proved to take part in stress responses and stress memory^23^. Chromatin-associated proteins, such as the high mobility group (HMG) proteins, including HMGA and HMGB subfamilies, function in gene expression regulation by changing chromatin structure^21,23^. In Arabidopsis, loss-of-function mutants of *HMGB5* negatively affected seed germination and plant growth under stress conditions^24^. Stemmer et al.^25^ found that maize HMGB2 was phosphorylated by protein kinase CK2 and phosphorylated HMGB2 was trans-localized to the cytosol and responded rapidly to the changing environments^26^. The up-regulated phosphorylation level of HMGA and HMGB2 in drought-primed plants under heat stress could positively contribute to improved heat tolerance in our study.

### Stress protection-related phosphorylated proteins associated with drought priming and priming-induced heat tolerance

Activating or upregulating stress protection systems provide safe guards for plants to endure long-term stress. Heat shock proteins are key components of stress protection systems by serving as molecular chaperones in assisting protein folding, transport, and degradation and participating in stress signaling along with other molecules, such as protein kinases and Ca^2+ 27^. The phosphorylation level of HSPs is essential for their function^20,27^. Post-translational phosphorylation of HSPs can be rapidly induced within minutes of exposure to heat and result in the subsequent accumulation of HSPs^28^. Lack of phosphorylation of HSP30 has been found to be related to the loss of function as a molecular chaperone due to the oligomeric structure changes^28^. Phosphorylation of HSP90s induces the release of substrate proteins and further regulates downstream gene expression^29^. The function of heat shock proteins in plant adaptation to drought and heat stress has been well characterized in many studies through transcriptome profiling or large-scale proteomics approach^30^, whereas little is known for the roles of HSP phosphorylation in priming-enhanced stress tolerance. In this study, phosphorylation levels of HSPs, including HSP23.6, HSP90-2, HSP90-1, and HSP70-4, were all up-regulated by drought priming and in response to heat stress. Interestingly, increased phosphorylation levels at one site (S58) of HSP23.6 and at one site (S302) of HSP70-4 were detected in drought-primed plants prior to and during heat stress, indicating that the increased phosphorylation level of specific sites in HSP23.6 and HSP70-4 due to drought priming was still maintained when plants were subsequently stressed by high temperature. Phosphorylation of HSP23.6 and HSP70-4 could play positive roles in drought priming-enhanced heat tolerance.

Dehydrins and aquaporins are also major components in plant stress defense systems^31,32^. Nodulin 26-like intrinsic proteins (NIP) group I functions as aquaglyceroporins mediating the flux of water and glycerol, while its phosphorylation level can be induced by water deficit resulting in enhanced transport activity^32^. Our results showed that the phosphorylation level of NIP4-2 was induced by drought priming and remained up-regulated in drought-primed plants under heat stress. Hernández-Sánchez et al.^31^ presented that aquaporin could interact with three members of the Arabidopsis dehydrin family and this PPI may contribute to the stabilization of the membranes. Recent study by Maszkowska et al.^33^ found that dehydrin ERD14 can be phosphorylated by SNF1‐related protein kinases 2 (SnRK2.10) and its phosphorylation might be involved in its nuclear import from cytoplasm in response to dehydration stress. The phosphorylation level of dehydrin family protein HIRD11 was up-regulated in response to drought priming in our study. The upregulation of phosphorylated aquaporins and dehydrins could facilitate drought priming and preconditioning plants with efficient water transport and dehydration protection for plant tolerance to later occurred heat stress.

### Stress perception- and signaling-related phosphorylated proteins associated with drought priming and priming-induced heat tolerance

Abscisic acid has been proven as a pivotal regulator of plant abiotic stress defense and regulates priming-induced stress memory^3^. The PYR/PYL/RCAR family of ABA receptors can trigger the loss function of phosphatase 2 C (PP2C), further activating faster transcription of stress-responsive genes^22^. In our study, two sites (S73, S75) of PYL5 exhibited upregulation in drought-primed plants. PYL5 plays a crucial role in ABA signal transduction pathway and overexpressing *PYL5* resulted in increased ABA sensitivity in Arabidopsis^34^. Wang et al.^35^ reported that phosphorylation of PYLs was positively related to growth recovery of plants from stress. Zinc-finger proteins are among the most common DNA-binding transcription regulators in plants, while CCCH-type zinc-finger protein has been reported involving in stress responses through its function as shuttle between the nucleus and cytoplasmic foci in eukaryotic cells^36^. Recent studies have demonstrated that CCCH tandem zinc-finger protein were positive regulators for ABA response, in part by upregulating ABA biosynthesis, enhancing ABA accumulation, and modulating ABA signaling pathways^37^. The phosphorylation level of zinc-finger CCCH domain protein (C3H8) was significantly increased in drought-primed plants under heat stress in our study.

Drought priming-induced stress responses signaling also involve phospholipid reprogramming, including increased content of lipid second messengers and long-chain unsaturated lipid classes^8^. Phosphoinositide-specific phospholipase C (PI-PLC) is an important enzyme generating two second messengers, inositol 1,4,5-trisphosphate (Ins(1,4,5)P3) and diacylglycerol (DAG) from phosphatidylinositol 4,5-bisphosphate (PI(4,5)P2). Ins(1,4,5)P3 induces Ca^2+^ release from intracellular stores, while DAG transiently triggers the activation of protein kinase C (PKC), and also can be subsequently phosphorylated to phosphatidic acid (PA) 38. It has been known that PLC contains multiple putative phosphorylation sites, and AtPLC2 was identified as one of the phosphorylated proteins in response to bacterial elicitor, however, the consequences of phosphorylation for PLC activity remain unknown^38,39^. By the phosphoproteomic approach, we detected an up-regulated phosphorylation level of PI-PLC6 at T322 site after drought priming. In addition, the phosphorylation level of other lipid-related proteins, such as LOX5 and LAG1, which involved in fatty acid biosynthetic and lipid metabolic processes, were also changed in response to drought priming. Since phosphorylation is an important PTM controlling enzymatic activity^38^, we suppose that drought priming may activate the phosphorylation of lipid metabolism-related proteins, regulate their activities, modulate the accumulation of lipid signaling molecules and unsaturated lipids, and further trigger the signaling pathway and protect plants in response to subsequent stresses.

### Protein phosphorylation motifs associated with drought priming and priming-induced heat tolerance

Identification of phosphorylation motifs is important to determine the binding of the kinase to its substrate and understand the signal transduction pathways^14^. Through phosphorylation motif analysis, we identified several distinct motifs regulated by drought priming. Sequence consensus analysis for phosphorylation sites showed [GS] motif was responsive to drought priming. [SP] motif was enriched from all heat stress treatments comparing to control temperature, which means that [SP] motif might be a common motif responsive to heat stress. This was in consistent with a previous study which showed that [SP] was shared in common under drought and heat stress treatments^40^. [SP] motif is a typical proline-directed motif, and is the potential substrate for the mitogen-activated protein kinase (MAPK) 41. Moreover, [DSD] motif was only found to be enriched in up-regulated phosphopeptides and [S..E] motif was found in both up- and down-regulated phosphopeptides in drought-primed plants under heat stress comparing with those under control temperature. These two motifs are both acidic motifs and can be recognized by casein kinase-II (CK-II), which appears to be associated with the regulation of cell growth, DNA damage repair, and other metabolic pathways through its signal transduction function to the cell cycle control^41,42^. These results indicated MAPKs could be important kinases for plant tolerance to heat stress, while CK-IIs might function in drought priming-enhanced heat tolerance.

**Fig. 6:**
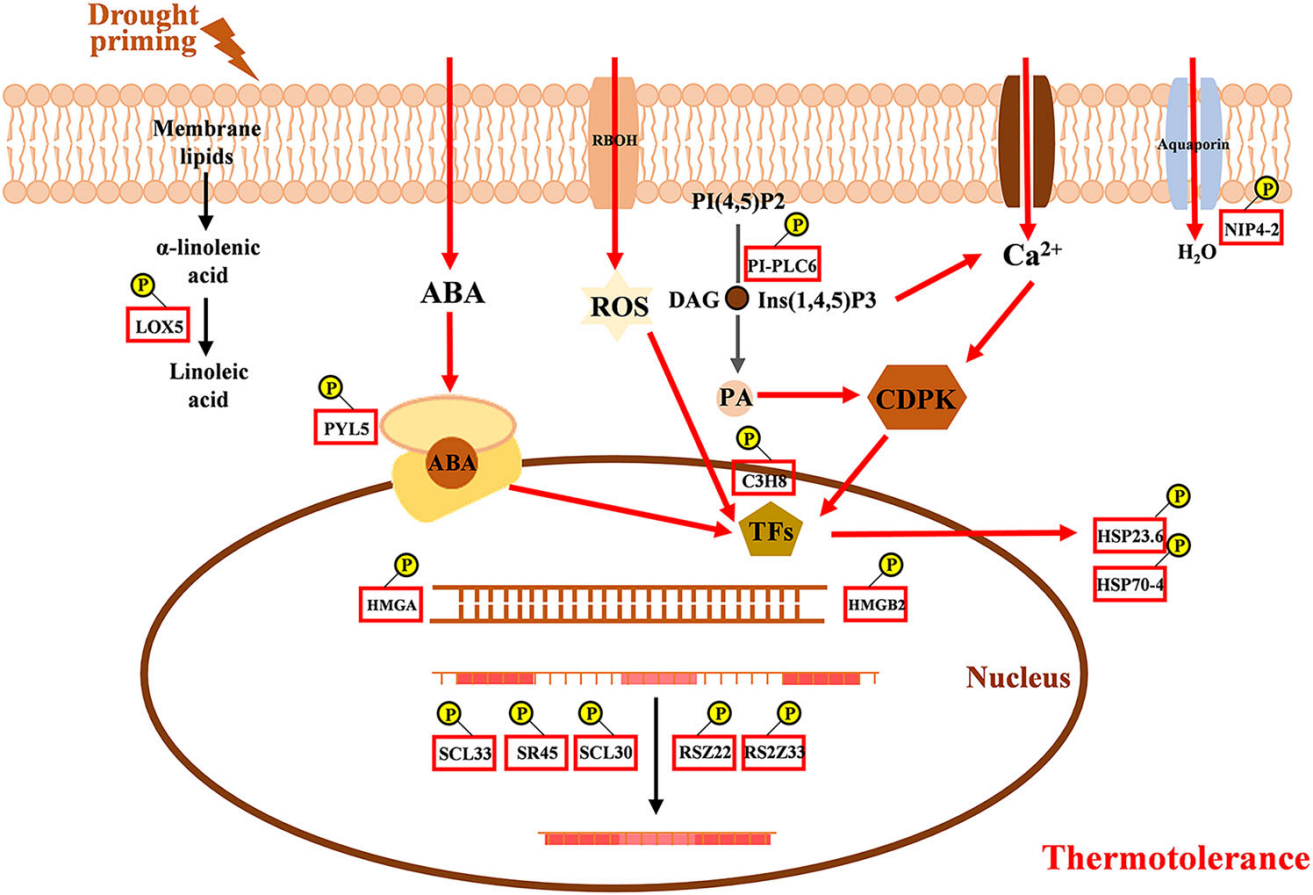
Schematic presentations of differentially regulated phosphoproteins involved in drought priming-enhanced thermotolerance. The differentially regulated phosphoproteins between D-H vs. ND-H in our study were used to construct the putative pathway involved in drought priming-enhanced thermotolerance. ND-H, non-drought priming + subsequent heat stress; D-H, drought priming + subsequent heat stress. Yellow balls represent phosphorylation. Proteins with red frames represent up-regulation while those with green frames represent down-regulation

In summary, this study identified phosphorylated proteins involved in RNA splicing, transcriptional control, stress protection/defense, and stress perception/signaling could be connected with drought priming-enhanced heat tolerance (Fig. 6). These results suggest the involvement of post-translational regulation of specific pathways in drought priming memory and cross-tolerance with heat stress. Furthermore, motif analysis detected several distinct motifs ([GS], [DSD] and [S..E]) regulated by drought priming and heat stress, which suggested that casein kinase-II and mitogen-activated protein kinase could be key protein kinases harnessed to drought priming-enhanced heat tolerance in plants.

## Materials and methods

### Plant materials and treatments

Tall fescue plants (cv. “Kentucky 31”) were established from seeds in 10-cm-inner-diameter, 40-cm-depth polyvinyl chloride (PVC) tubes with sand soil mixtures (sand and loamy soil, 1:2, V/V) and maintained in a greenhouse at Rutgers University in New Brunswick, NJ. Plants were irrigated daily, fertilized with half-strength Hoagland’s nutrient solution^43^ once weekly, and kept at approximately 10 cm canopy height by trimming every three days during establishment.

Following 2 months establishment period, tall fescue plants were then moved into environmental growth chambers and acclimated for one week before initiation of treatments. The growth chambers were controlled to maintain at a temperature of 23/18 °C day/night, 14 hr light/10 h darkness photoperiod, 60% relative humidity, and 650 µmol m^−2^ s^−1^ photosynthetically active radiation.

The experimental treatments and design followed the description in our previous study^8^. Non-drought primed plants (ND-) were supplied with adequate water every day, while drought-primed plants (D-) were withhold from irrigation for 8 days till soil volumetric water content (SVWC) declined to about 10%, which was approximately 50% of the full field capacity. After drought priming, all plants were irrigated sufficiently and then subjected to heat stress (38/33 °C, day/night) or optimal air temperature (23/18 °C) conditions for 25 days. The experiment included four treatments: (1) non-drought priming followed with optimal temperature (ND-C), (2) drought priming followed with optimal temperature (D-C), (3) drought-priming followed with heat stress (D-H), and (4) non-drought priming followed with heat stress (ND-H). Each treatment was replicated three times and plants were randomly placed in growth chambers and rotated every five days between the chambers to minimize any potential effects caused by growth chambers.

To identify changes in protein phosphorylation patterns resulting from drought priming and heat stress, leaves from tall fescue plants grown under different treatments at the end of drought priming (D-, ND-) and at the end of heat stress (D-C, ND-C, D-H, ND-H) were collected. Leaf samples were taken from all treatments, immediately immersed in liquid nitrogen, and transferred to storage at -80 °C. The experimental design and phosphoproteomic workflow were shown in Fig. S3.

### Protein extraction

Frozen tall fescue leaf samples (about 100 mg) were quickly ground into fine and uniform powder in liquid nitrogen and then homogenized in 1 mL phenol extraction buffer, after that 1 mL saturated phenol with Tris-HCl (pH 7.5) was added. After several times shake, the mixture was kept at 4 °C for 30 min. The upper phenolic phase was separated from the aqueous phase by centrifugation at 7100 *g* at 4 °C for 10 min, transferred to a fresh tube and mixed with five volumes of pre-cold 0.1 M ammonium acetate-methanol. After being kept at −20 °C overnight, the mixture was centrifuged at 12,000 *g* for 10 min at 4 °C to pellet precipitated protein. For wash step, the pellet was resuspended twice with pre-cold methanol and twice with ice-cold acetone. Following another round of centrifugation, the pellet was collected, air-dried and resuspended with 300 μL lysate solution. After incubation of 3 hr at room temperature, the solution was centrifuged to remove any insoluble fraction and the resulting supernatant contained the total extractable protein. The total protein concentrations were quantified by bicinchoninic acid assay^44^.

### Protein digestion and tandem mass tags (TMT) labeling

One hundred microgram protein for each sample was used for trypsin digestion following filter-aided sample preparation method^45^. Protein samples were transferred into new 10 K ultrafiltration tubes, and the flow-through was discarded after centrifugation. Protein reduction was done at 60 °C for 1 hr after resuspending the protein with 120 μL reducing agent (10 mM dithiothreitol, 8 M urea, 0.1 M triethylammonium bicarbonate (TEAB), pH 8.0). Alkylation was performed through the addition of iodoacetamide to a final concentration of 50 mM. The filter units were washed twice with 100 μL 0.3 M TEAB. The sample was reconstituted in 100 μL 0.3 M TEAB and the peptide fragments generated by digestion with trypsin (1 μg μL^−1^) were collected from centrifugation at 12000 rpm for 20 min. The samples were vacuum freeze dried, resuspended with 100 μL 0.2 M TEAB and then 40 μL of which was transferred into a new Eppendorf tube for TMT labeling.

The TMT labeling was performed according to the manufacturer’s instructions (Thermo Fisher Scientific, Asheville, NC, USA). After adding 41 μL anhydrous acetonitrile, each vial was vortexed for 5 min to mix well and then centrifuged briefly to spin down the solution. Then, 41 μL of TMT label reagent was added into each sample and the reaction was incubated at room temperature for 1 hr. Finally, the labeling reaction was quenched by adding 8 μL of 5% hydroxylamine. The labeled peptides were lyophilized and stored at -80 °C.

### Phosphorylated peptide enrichment

The enrichment of phosphopeptides with titanium dioxide (TiO_2_) beads was performed following the procedure of Jersie-Christensen et al.^46^ with minor modifications. The labeled phosphopeptides were resuspended in 100 μL loading buffer (Glutamate saturated solution with 2% TFA 60% ACN). The peptide lysate and TiO_2_ beads (1:4, m/m) were incubated in a reaction tube and vortexed for 15 min. After incubation, the sediment was collected by centrifugation for 1 min at 6000 rpm under room temperature, and washed twice with 400 μL wash buffer 1 (0.5% TFA, 50% ACN), and subsequently with wash buffer 2 (0.1% TFA, 50% acetonitrile). The peptide-bound beads were pelleted and the peptides are finally eluted with 100 μL 10% NH_4_OH and vacuum concentrated for further analysis.

### Liquid chromatography and mass spectrometry (LC-MS/MS) analysis

The enriched phosphopeptide samples were analyzed by LC-MS/MS on a Nanospray Flex source coupled to a Q-Exactive mass spectrometer (Thermo Fisher Scientific, USA). Peptide mixtures were firstly loaded onto a C18 nano-trap column (Acclaim PepMap100, 200 mm length × 100 μm inside diameter, Thermo Fisher Scientific) and then separated on an analytical column (Acclaim PepMap RSLC, 150 mm length × 75 μm inside diameter) on an EASY-nLCTM 1200 system (Thermo Fisher Scientific). The mobile phases consisted of 0.1% formic acid (A) and 0.1% formic acid and 80% ACN (B) in a three-step linear gradient of 0–20% solution B over 47 min, 20–40% solution B over 26 min, and 40–90% solution B over 4 min.

In positive ion mode, the mass spectrometry full scan range had a mass/charge ratio (m/z) of 300-1600 with a mass resolution of 70,000, and the twelve most intense peaks in MS were selected and further fragmented with higher-energy collisional dissociation with a normalized collision energy of 30. MS/MS spectra were acquired at 17500 resolution with a max injection time of 80 ms, and the dynamic exclusion time was set to 30 s.

### Database search and data analysis

The resulting mass spectrometric data were queried against the UniProt database^47^ and a cDNA library kept by our lab using Proteome Discoverer version 2.2 (Thermo Fisher Scientific). Trypsin/P was set as enzyme digestion mode, along with cysteine alkylation as the fixed modification in the database searching. The quantitative method was set as TMT-6plex, and the value of 0.01 was specified as the false discovery rate. The significant fold-change cut was 1.3-fold at the 95% confidence interval for differential proteins between treatments.

### Bioinformatic analysis

The functional annotation of phosphorylated proteins was characterized using UniProt (http://www.uniprot.org/) and GO (http://www.geneontology.org/). The amino acid sequences of identified UniProt IDs were submitted to blast against the Arabidopsis Information Resource (http://www.arabidopsis.org) to obtain more functional annotation, and the correspondent Arabidopsis IDs were used for further analysis. Protein functions were categorized based on MapMan ontology and the subcellular location was predicted from the consensus location available from SUBA3^48,49^. The phosphorylation motifs of PTM sites were analyzed by using the Motif-X algorithm in MoMo Modification Motifs (MEME Suite5.1.0, http://meme-suite.org/tools/momo) with *P*-value threshold of 10^−6 ^^50^.

## Supplementary information

Supplementary Figure S1- S3

Supplementary Table S1

Supplementary Table S2- S4

Supplementary Table S5
