# Supplementary material for: Protein phosphorylation associated with drought priming-enhanced heat tolerance in a temperate grass species
Source: Hortic Res. 2020 Dec 1;7:207. doi: 10.1038/s41438-020-00440-8 (PMC7705721; doi:10.1038/s41438-020-00440-8)

Fig. S1 Mass spectrometry analysis of phosphoproteomic changes of tall fescue leaves in response to drought priming and subsequent heat stress.

A) The number of identified and differentially expressed phosphopeptides and phosphosites; B) Distribution of assigned phospho-amino acid residues; C) Distribution of singly- and multiply-phosphorylated peptides.


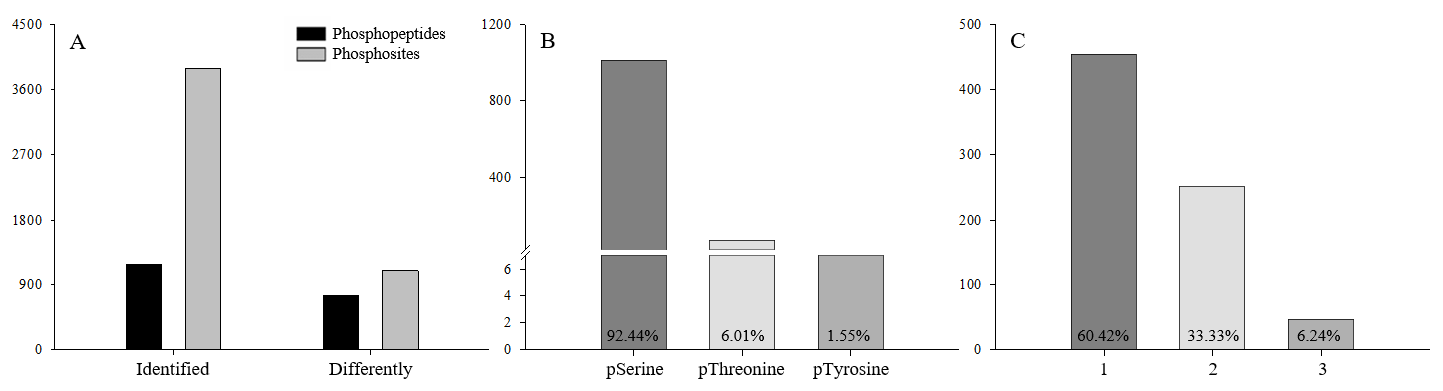

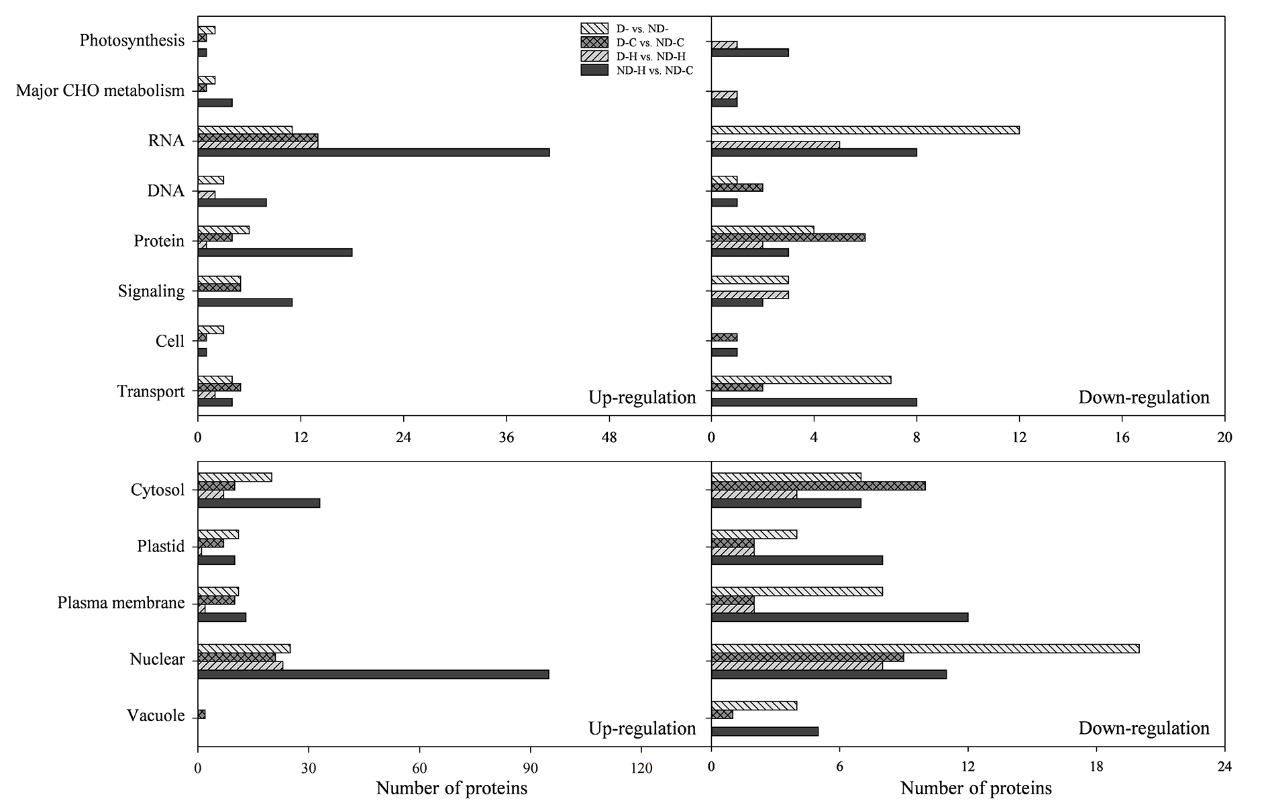


Fig. S2 Number of differently expressed phosphoproteins according to functional categories and subcellular compartments by MapMan

Note: ND-, no drought priming; D-, drought priming. ND-C, non-drought priming + control temperature; D-C, drought priming + control temperature; ND-H, non-drought priming + subsequent heat stress; D-H, drought priming + subsequent heat stress.

Fig. S3 The experimental design and workflow for quantitative phosphoproteomic analysis of tall fescue leaves in response to drought priming and subsequent heat stress

Note: ND-, no drought priming; D-, drought priming. ND-C, non-drought priming + control temperature; D-C, drought priming + control temperature; ND-H, non-drought priming + subsequent heat stress; D-H, drought priming + subsequent heat stress. Tall fescue leaves were collected at the end of drought priming and heat stress. Total proteins were extracted, digested using trypsin, labeled with TMT, and then enriched using titanium dioxide beads (TiO_2_). The phosphopeptide samples were analyzed by LC-MS/MS and the data were processed with bioinformatic tools.


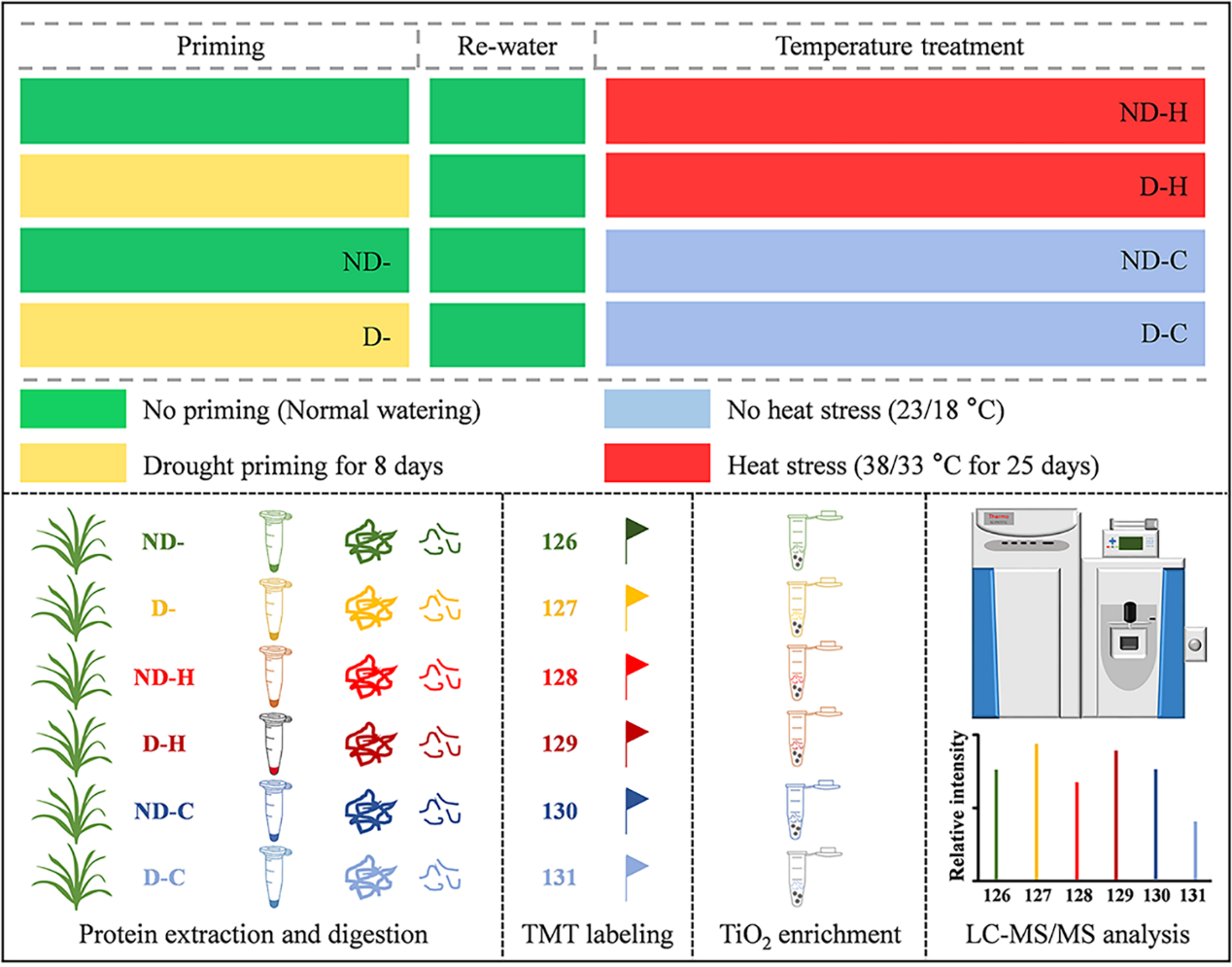

Supplement: Supplementary file 1 — Supplementary Figure S1- S3 [file 41438_2020_440_MOESM1_ESM.docx]
