# Supplementary material for: Protein phosphorylation associated with drought priming-enhanced heat tolerance in a temperate grass species
Source: Hortic Res. 2020 Dec 1;7:207. doi: 10.1038/s41438-020-00440-8 (PMC7705721; doi:10.1038/s41438-020-00440-8)
Supplement: Supplementary file 3 — Supplementary Table S2- S4 [file 41438_2020_440_MOESM3_ESM.docx]

Table S2 Details of significantly changed phosphopeptides in response to drought priming in tall fescue leaves

| Protein accession | Description | Locus number | Sequence | D- vs. ND- | Adjusted P-value | P-site in protein |
| --- | --- | --- | --- | --- | --- | --- |
| Up-regulated phosphopeptids | | | | | | |
| CL6257.Contig2_All | Serine/threonine-protein kinase STN7, chloroplastic | AT1G68830 | TINE**S**(ph)MGELTSQSK | 1.35 | 1.71E-02 | S541 |
| Unigene24022_All | Ribulose bisphosphate carboxylase large chain | ATCG00490 | AIKFEFEPVD**T**(ph)IDN | 1.83 | 1.32E-04 | T474 |
| CL17602.Contig3_All | Sucrose synthase 3 | AT4G02280 | LDRNP**S**(ph)IR | 1.47 | 3.46E-03 | S31 |
| CL5834.Contig3_All | Sucrose phosphate synthase 4F | AT4G10120 | IN**S**(ph)EVRLV**S**(ph)DDEEEQTK | 1.71 | 8.83E-03 | S176; S182 |
| Unigene15217_All | Fructose-2,6-bisphosphatase | AT1G07110 | SFSAC**S**(ph)LA**S**(ph)GLNFGSTK | 2.29 | 4.85E-04 | S337; S340 |
| CL8978.Contig4_All | Beta carbonic anhydrase 2, chloroplastic | AT5G14740 | DGADD**S**(ph)FHFVEDWVR | 1.59 | 6.89E-03 | S158 |
| CL6787.Contig1_All | Aluminum induced protein with YGL and LRDR motifs | AT5G43830 | VDSQGQMCG**S**(ph)TFK | 1.97 | 5.10E-05 | S251 |
| Unigene34796_All | 23.6 kDa heat shock protein, mitochondrial | AT4G25200 | EE**S**(ph)DDDSRR | 2.12 | 2.56E-03 | S58 |
| CL14785.Contig1_All | Heat shock 70 kDa protein 4 | AT3G12580 | LMPEPTAVALLYAQQQQQLLHDNMG**S**(ph)GIEK | 1.88 | 1.04E-03 | S302 |
| CL4294.Contig1_All | Heat shock 70 kDa protein 14 | AT1G79930 | SVPMDTDAK**S**(ph)PSK | 1.86 | 4.93E-04 | S558 |
| CL10895.Contig9_All | Heat shock protein 90-1 | AT5G52640 | EI**S**(ph)DDEDEDDAAEK | 3.25 | 2.77E-04 | S279 |
| CL10895.Contig10_All | Heat shock protein 90-2 | AT5G56030 | TTEKEI**S**(ph)DDEDEDDAADK | 3.12 | 4.54E-03 | S269 |
| CL14676.Contig3_All | Dehydrin HIRD11 | AT1G54410 | KHGEGHKDDGHSSS**S**(ph)SDSD | 4.46 | 5.10E-05 | S95 |
| CL14676.Contig3_All | Dehydrin HIRD11 | AT1G54410 | KHGEGHKDDGHSSSS**S**(ph)DSD | 4.25 | 8.03E-05 | S96 |
| CL14676.Contig4_All | Dehydrin HIRD11 | AT1G54410 | ITGEHGDK**S**(ph)**S**(ph)DDHKEK | 1.75 | 2.85E-03 | S64; S65 |
| CL14676.Contig4_All | Dehydrin HIRD11 | AT1G54410 | ITGEHGDK**S**(ph)SDDHK | 1.77 | 6.22E-03 | S64 |
| CL840.Contig1_All | Oleosin-B3-like protein | AT1G13930 | Q**Y**(ph)SSGGAEK | 1.55 | 7.10E-03 | Y75 |
| CL15119.Contig3_All | Probable glutathione peroxidase 4 | AT2G48150 | SD**S**(ph)QKGPEGESIPAR | 1.49 | 3.21E-03 | S257 |
| CL15119.Contig3_All | Probable glutathione peroxidase 4 | AT2G48150 | SG**S**(ph)QKAPEGENIPAR | 1.67 | 2.02E-03 | S212 |
| CL15370.Contig3_All | AMP deaminase | AT2G38280 | VGLIRPN**S**(ph)PK**S**(ph)PVASASTFESIEGPDEDVASK | 1.48 | 1.60E-02 | S184; S187 |
| CL15370.Contig2_All | AMP deaminase | AT2G38280 | VGLIRPN**S**(ph)PK**S**(ph)PVA**S**(ph)ASTFESIEGSDEDVASK | 1.40 | 2.76E-02 | S184; S187; S191 |
| CL3317.Contig1_All | Serine/arginine-rich splicing factor SR45 | AT1G16610 | RH**S**(ph)R**S**(ph)PPPR | 1.38 | 2.43E-03 | S327; S329 |
| CL6548.Contig5_All | Dynamin-related protein 2A | AT1G10290 | HVDA**S**(ph)DDDVDK | 1.33 | 3.02E-04 | S523 |
| CL4046.Contig3_All | Hydroxysteroid dehydrogenase 5 | AT4G10020 | HAQEDGNLAR**S**(ph)G**S**(ph)PPAR | 1.32 | 1.73E-03 | S293; S295 |
| CL6723.Contig3_All | Splicing factor U2af small subunit A | AT1G27650 | SR**S**(ph)P**S**(ph)PQHR | 1.6 | 1.12E-03 | S250; S252 |
| CL6723.Contig3_All | Splicing factor U2af small subunit A | AT1G27650 | AR**S**(ph)PVREN**S**(ph)EER | 1.38 | 1.26E-02 | S316; S322 |
| CL3199.Contig4_All | Ethylene-responsive transcription factor RAP2-1 | AT1G46768 | AKNPDLNREP**T**(ph)PD**T**(ph)DDDE | 1.47 | 1.72E-03 | T213; T216 |
| CL5517.Contig7_All | Serine/arginine-rich SC35-like splicing factor SCL30 | AT3G55460 | R**Y**(ph)**S**(ph)PPHR**S**(ph)PLR | 1.31 | 6.16E-03 | Y40; S41; S46 |
| Unigene29705_All | Chromatin remodeling protein SHL | AT4G39100 | TAEN**S**(ph)HEATAQ**S**(ph)DEKPVESK | 1.34 | 1.39E-03 | S199; S206 |
| CL7594.Contig7_All | RNA-binding (RRM/RBD/RNP motifs) family protein | AT5G55670 | **S**(ph)R**S**(ph)REVDHSK | 1.47 | 4.98E-04 | S251; S253 |
| CL6783.Contig3_All | Serine/arginine-rich splicing factor RS41 | AT5G52040 | SGGDDEY**S**(ph)GGGYK | 1.38 | 1.12E-03 | S272 |
| CL6783.Contig5_All | Serine/arginine-rich splicing factor RS31A | AT2G46610 | SGTDDYDR**S**(ph)EGR | 1.63 | 1.03E-03 | S87 |
| CL6783.Contig5_All | Serine/arginine-rich splicing factor RS31A | AT2G46610 | STGDDEY**S**(ph)GGAGYQK | 1.46 | 1.86E-03 | S97 |
| CL8553.Contig4_All | Methyl-CpG-binding domain-containing protein 10 | AT1G15340 | VFD**S**(ph)PEGEK**T**(ph)PKR | 1.30 | 7.51E-03 | S123; T129 |
| CL4905.Contig1_All | SAP domain-containing protein | AT4G39680 | SFGR**S**(ph)D**S**(ph)TASGDSPK | 1.36 | 2.05E-03 | S491; S493 |
| CL5702.Contig5_All | HMG-Y-related protein A | AT1G14900 | AKG**S**(ph)PAGK**S**(ph)PAPAPK | 1.36 | 4.59E-03 | S127; S132 |
| CL10322.Contig1_All | DNA-damage-repair/toleration protein DRT111, chloroplastic | AT1G30480 | QRPPPPSPSLSQQQ**S**(ph)R | 1.61 | 1.15E-03 | S26 |
| CL12933.Contig3_All | Methyl-CpG-binding domain protein 4-like protein | AT3G07930 | II**T**(ph)PHFNSLASEELG**T**(ph)AYK | 1.37 | 4.90E-04 | T63; T76 |
| CL1335.Contig2_All | Metacaspase-4 | AT1G79340 | SQHGGEDQDE**S**(ph)DEQPTGDGHTK | 1.43 | 5.00E-03 | S351 |
| CL2686.Contig3_All | Translation initiation factor IF3-2, chloroplastic | AT2G24060 | YSSYG**S**(ph)DEDDDEDGGGGR | 1.76 | 1.12E-03 | S67 |
| CL2686.Contig2_All | Translation initiation factor IF3-2, chloroplastic | AT2G24060 | YSSYG**S**(ph)DEDDDEEGGGGR | 1.70 | 6.56E-03 | S67 |
| CL8960.Contig2_All | Eukaryotic translation initiation factor 4B1 | AT3G26400 | ERG**S**(ph)FGGGGSSDR | 1.41 | 3.22E-03 | S560 |
| CL15595.Contig3_All | Peptide methionine sulfoxide reductase A4, chloroplastic | AT4G25130 | LGLGGGG**S**(ph)PR | 1.44 | 1.11E-03 | S111 |
| CL1562.Contig3_All | Translocase of chloroplast 159, chloroplastic | AT4G02510 | VVEKEEVKGD**S**(ph)GDGSK | 1.47 | 3.40E-03 | S50 |
| CL6749.Contig2_All | Calcium-dependent protein kinase 7 | AT5G12480 | DG**S**(ph)INDDDRK | 1.57 | 5.84E-04 | S525 |
| CL7021.Contig2_All | Sodium/calcium exchanger NCL | AT1G53210 | VPASGAY**S**(ph)NK | 1.52 | 4.37E-04 | S387 |
| CL20158.Contig2_All | Phototropin-1 | AT3G45780 | SSGGGG**S**(ph)AR**S**(ph)SSDDK | 1.40 | 1.77E-02 | S104; S107 |
| CL7021.Contig1_All | Sodium/calcium exchanger NCL | AT1G53210 | NKVPASGAN**S**(ph)NK | 1.67 | 5.14E-02 | S387 |
| CL7939.Contig2_All | Phosphoinositide phospholipase C 6 | AT2G40116 | AGG**T**(ph)IKDTDGKGGDDDAAWGK | 1.67 | 2.47E-02 | T322 |
| Unigene27307_All | Protein PLASTID MOVEMENT IMPAIRED 1 | AT1G42550 | KQ**S**(ph)K**T**(ph)**S**(ph)FSITSPK | 2.40 | 2.68E-04 | S106; S108, S109 |
| CL18724.Contig2_All | Regulator of chromosome condensation (RCC1) family protein | AT3G55580 | T**S**(ph)SGAASGPSESR | 1.83 | 1.11E-02 | S206 |
| CL15237.Contig2_All | Microfibrillar-associated protein-like protein | AT4G08580 | HDGDGDR**S**(ph)PKDDPR | 2.00 | 7.57E-04 | S69 |
| CL4685.Contig3_All | Late embryogenesis abundant protein 7 | AT1G52690 | AE**S**(ph)PVAPK | 1.60 | 9.34E-04 | S150 |
| CL9184.Contig2_All | Magnesium transporter MRS2-11, chloroplastic | AT5G22830 | AVAKEEEVEED**S**(ph)EEEER | 1.37 | 5.82E-03 | S107 |
| Unigene55432_All | Amino acid transporter AVT1A | AT2G41190 | SSLA**S**(ph)DSK | 1.63 | 2.02E-03 | S113 |
| CL3378.Contig7_All | Probable aquaporin NIP4-2 | AT5G37820 | RLQ**S**(ph)QR**S**(ph)AADDFDHV | 1.66 | 4.98E-04 | S286; S289 |
| CL12882.Contig3_All | Nitrate transporter 1.3 | AT3G21670 | AGDEDG**S**(ph)TGKEMDSVLVVK | 1.47 | 1.83E-02 | S574 |
| CL856.Contig1_All | Serine/arginine-rich splicing factor SR45a | AT1G07350 | RR**S**(ph)Y**S**(ph)PYGR | 1.71 | 3.42E-03 | S241; S243 |
| CL8070.Contig3_All | Pre-mRNA-splicing factor | AT3G49601 | DTRPISEAD**S**(ph)E**S**(ph)DHGKK | 1.31 | 6.89E-03 | S335; S337 |
| CL1844.Contig16_All | Putative serine/threonine protein kinase | AT1G11440 | HDEDND**S**(ph)DDEKK | 1.61 | 1.36E-04 | S382 |
| CL2208.Contig2_All | Ninja-family protein AFP2 | AT1G13740 | TS**S**(ph)LPTETEEER | 2.51 | 5.90E-03 | S91 |
| CL15451.Contig7_All | DNA polymerase epsilon catalytic subunit A | AT1G19530 | GHEETAEGDAAADDTGG**S**(ph)PGK | 1.65 | 5.85E-03 | S95 |
| Unigene51511_All | NPL4-like protein 2 | AT2G47970 | ALAPAG**S**(ph)FGK | 1.73 | 7.39E-05 | S244 |
| CL8070.Contig3_All | Pre-mRNA-splicing factor | AT3G49601 | HD**S**(ph)EDEK | 1.34 | 1.63E-02 | S488 |
| Unigene32467_All | Protein CHUP1, chloroplastic | AT3G25690 | DLNR**T**(ph)L**S**(ph)PK | 1.59 | 2.99E-03 | T122; S124 |
| CL14330.Contig1_All | ABI five-binding protein 3 | AT3G29575 | FGTDR**S**(ph)PDAK | 1.54 | 4.21E-03 | S58 |
| CL8368.Contig2_All | GCIP-interacting family protein | AT2G16860 | STSSSEDG**S**(ph)VK | 1.44 | 2.38E-02 | S255 |
| CL6783.Contig3_All | Putative glycine-rich cell wall protein | AT4G36230 | **S**(ph)GDDYDNK | 1.40 | 4.30E-04 | S31 |
| CL12766.Contig1_All | Thylakoid soluble phosphoprotein | AT3G47070 | KAGG**T**(ph)TSK | 1.39 | 1.01E-04 | T136 |
| Down-regulated phosphopeptides | | | | | | |
| CL19104.Contig4_All | Phosphoenolpyruvate carboxykinase | AT4G37870 | **S**(ph)AP**T**(ph)TPIK | 0.17 | 1.86E-03 | S87; T90 |
| CL2395.Contig4_All | LAG1 longevity assurance homolog 3 | AT1G13580 | GHVGDDIR**S**(ph)D**S**(ph)ESEEEHED | 0.15 | 1.02E-02 | S375; S377 |
| CL840.Contig1_All | F16A14.27 (Oleosin-B3-like protein) | AT1G13930 | QY**S**(ph)**S**(ph)GGAEKEK | 0.16 | 5.74E-02 | S76; S77 |
| Unigene46642_All | Allantoinase | AT4G04955 | IPDVA**S**(ph)DDELDGELDPR | 0.22 | 5.67E-02 | S274 |
| CL6806.Contig4_All | Dynamin-related protein 2B | AT1G59610 | AAVSSYSND**S**(ph)PEAG**S**(ph)PRTP**S**(ph)RPGEDWR | 0.19 | 1.07E-03 | S895; S900; S905 |
| CL11990.Contig6_All | Glycosyl hydrolases family 31 protein | AT5G11720 | EQDEEEET**S**(ph)DTEKEEK | 0.20 | 8.46E-05 | S3088 |
| CL14546.Contig4_All | ATG8-interacting protein 1 | AT3G55460 | GSQYAV**S**(ph)DTGDDDRDEEK | 0.23 | 3.06E-03 | S122 |
| Unigene50674_All | Nuclear protein-like (Splicing factor) | AT5G64270 | LPGGLV**T**(ph)P**T**(ph)PK | 0.16 | 1.53E-02 | T310; T312 |
| Unigene50674_All | Nuclear protein-like (Splicing factor) | AT5G64270 | LLA**T**(ph)P**T**(ph)PL**T**(ph)TPLYNIPEENR | 0.18 | 7.08E-03 | T424; T426; T429 |
| CL15571.Contig1_All | DEAD-box ATP-dependent RNA helicase 3, chloroplastic | AT5G26742 | LGLGG**S**(ph)DDEDEDEFG**S**(ph)DQEGAAGEALQGDADELAISR | 0.17 | 8.76E-02 | S54; S64 |
| CL2001.Contig3_All | HEAT repeat-containing protein | AT5G01400 | LLEAQNIINQGDSVDHTANDS**T**(ph)EH**T**(ph)ANAAR | 0.14 | 1.04E-02 | T326; T329 |
| CL22152.Contig4_All | DNA-directed RNA polymerase I subunit 1 | AT3G57660 | YKPDNEDDPAE**S**(ph)GGE**S**(ph)EVEDEGHM**S**(ph)DSGNK | 0.21 | 3.17E-02 | S1167; S1171; S1180 |
| CL250.Contig9_All | Histone deacetylase HDT3 | AT5G03740 | VFQPAEGDEMDFDDEDE**S**(ph)EGEEK | 0.22 | 7.73E-03 | S150 |
| Unigene20959_All | High mobility group B protein 2 | AT1G20693 | AAPAAKDEDEDEEE**S**(ph)DKSK | 0.22 | 7.05E-03 | S140 |
| CL4339.Contig1_All | Repressor of RNA polymerase III transcription | AT5G13240 | FAGDDFLAGVL**S**(ph)EGEEDALIDMDI | 0.23 | 3.84E-02 | S211 |
| CL2360.Contig3_All | MIF4G domain-containing protein | AT1G80930 | A**S**(ph)DDERDDRR | 0.41 | 9.55E-03 | S146 |
| Unigene33629_All | Heterogeneous nuclear ribonucleoprotein Q | AT4G00830 | AA**S**(ph)QGDYDEQDR | 0.16 | 3.06E-02 | S7 |
| CL18551.Contig1_All | RNA-binding family protein | AT3G07810 | DDQQAL**S**(ph)K | 0.27 | 4.85E-04 | S90 |
| CL13457.Contig2_All | Tetratricopeptide repeat -like superfamily protein | AT5G28740 | AESSTEQAAANNEDIELPDEE**S**(ph)DEEADVQIEER | 0.16 | 5.02E-03 | S911 |
| Unigene590_All | 60S acidic ribosomal protein P2-3 | AT3G28500 | EE**S**(ph)DDDMGFSLFD | 0.22 | 3.20E-03 | S135 |
| Unigene24129_All | Kinase superfamily with octicosapeptide/ Phox/Bem1p domain-containing protein | AT5G57610 | VKQH**T**(ph)LV**S**(ph)GGVR | 0.53 | 2.51E-03 | T996; S999 |
| CL492.Contig4_All | Outer arm dynein light chain 1 protein | AT4G03260 | ADYL**S**(ph)GDETNR**S**(ph)K | 0.18 | 2.52E-03 | S418; S425 |
| CL7479.Contig5_All | Kinase superfamily with octicosapeptide/ Phox/Bem1p domain-containing protein | AT3G24715 | RN**T**(ph)LV**S**(ph)GGVR | 0.32 | 1.60E-03 | T1005; S1008 |
| CL8966.Contig2_All | Probable inactive receptor kinase | AT1G27190 | DV**S**(ph)EQFDEFPLAYNK | 0.17 | 2.42E-02 | S623 |
| CL9411.Contig2_All | Calcium-binding EF hand family protein | AT1G20760 | FGHDS**S**(ph)PTK | 0.18 | 3.88E-02 | S965 |
| Unigene535_All | Calmodulin-like protein 25 | AT1G24620 | DAAADR**S**(ph)PPR**S**(ph)PTR | 0.21 | 6.40E-03 | S33; S37 |
| CL19970.Contig8_All | ATPase 2, plasma membrane-type | AT4G30190 | GLDIDTINQNY**T**(ph)V | 0.30 | 2.68E-04 | T955 |
| CL3521.Contig2_All | Vacuolar cation/proton exchanger 3 | AT3G51860 | TAHGM**S**(ph)**S**(ph)SSLR | 0.17 | 3.22E-03 | S46, S47 |
| CL1551.Contig1_All | Sodium/hydrogen exchanger 7 | AT2G01980 | AIAEEDN**S**(ph)**S**(ph)DE**S**(ph)VEEEIIVR | 0.29 | 3.19E-02 | S1140; S1141; S1144 |
| CL2267.Contig7_All | Monosaccharide-sensing protein 2 | AT4G35300 | IYLHQEGVPD**S**(ph)R | 0.17 | 9.66E-03 | S447 |
| CL2267.Contig7_All | Monosaccharide-sensing protein 2 | AT4G35300 | GPSMLGSVFSLA**S**(ph)R | 0.21 | 9.19E-04 | S286 |
| Unigene957_All | Sugar transporter MSSP2 | AT4G35300 | RG**S**(ph)IL**S**(ph)MPGGDVPPGGEYIQAAALVSQPALYSK | 0.54 | 2.02E-03 | S456; S459 |
| Unigene50821_All | Protein PIN-LIKES 7 | AT5G65980 | SIQCPAD**S**(ph)DEEHLEQFK | 0.33 | 2.48E-03 | S190 |
| CL12272.Contig6_All | Sas10/U3 ribonucleoprotein (Utp) family protein | AT2G43650 | VRGEDDELPQDGDYAEQKPDQ**S**(ph)**S**(ph)DEDEFYK | 0.19 | 2.47E-02 | S127; S128 |
| Unigene24450_All | Membrane protein of ER body-like protein | AT4G27870 | SGPEEEETNETP**S**(ph)PK | 0.27 | 6.81E-03 | S112 |
| CL14203.Contig2_All | Cwf15 / Cwc15 cell cycle control family protein | AT3G13200 | EIDADD**S**(ph)DVEPR**S**(ph)DDE**S**(ph)DDDDDDDDTEALMAELER | 0.23 | 2.50E-02 | S112; S118; S122 |
| Unigene13640_All | Divalent ion symporter (T7I23.21 protein) | AT1G02260 | SVIIEDAPP**S**(ph)PPD**S**(ph)VAADGEKDKELPEVVEK | 0.25 | 9.65E-04 | S337; S341 |
| Unigene29452_All | Tetratricopeptide repeat -like superfamily protein | AT1G01320 | AEVNIN**S**(ph)PR | 0.15 | 9.76E-04 | S910 |
| CL755.Contig19_All | Expressed protein | AT2G15270 | KSEEM**S**(ph)DEDNQGEEDAEEK | 0.28 | 4.27E-04 | S529 |
| CL5232.Contig2_All | Bromodomain protein (DUF761) | AT2G26110 | DRPSRPLVVAEPEEPV**S**(ph)EEEAEEAGGGEVDAR | 0.29 | 2.92E-03 | S227 |
| CL13660.Contig1_All | ATG8-interacting protein 1 | AT2G45980 | AEDDGHGSAVHDDDDD**S**(ph)QDR | 0.27 | 3.17E-02 | S145 |
| CL14546.Contig3_All | ATG8-interacting protein 1 | AT2G45980 | GSQYAV**S**(ph)DTGDDRDEEK | 0.17 | 4.37E-03 | S115 |
| CL13646.Contig1_All | Protamine P1 family protein | AT5G03110 | AAVPEDEP**S**(ph)SPK | 0.17 | 5.85E-03 | S154 |
| Unigene18546_All | Transcriptional regulator | AT4G31880 | L**S**(ph)ERTI**S**(ph)DELPQESSK | 0.15 | 2.09E-03 | S317; S322 |
| CL1964.Contig4_All | Small acidic-like protein | AT3G02220 | EGDLFPVASLDEYAEQAIQNDD**S**(ph)DEEEPDFVEG | 0.17 | 2.82E-02 | S215 |
| Unigene53956_All | Tracheary element differentiation-related 6 | AT1G43790 | IEVL**S**(ph)EDEDVRFEEADNKEEASEK | 0.56 | 8.97E-05 | S101 |

Note: ND-, no drought priming; D-, drought priming. Locus number are the correspondent Arabidopsis IDs blasted against The Arabidopsis Information Resource (TAIR) using sequences of identified UniProt IDs.

Table S3 Details of significantly changed phosphopeptides in response to drought priming under heat stress in tall fescue leaves

| Protein accession | Description | Locus number | Sequence | D-H vs. ND-H | Adjusted P-value | P-site in protein |
| --- | --- | --- | --- | --- | --- | --- |
| Up-regulated phosphopeptides | | | | | | |
| Unigene4466_All | Pyruvate dehydrogenase E1 component subunit alpha-1, mitochondrial | AT1G59900 | YHGH**S**(ph)MSDPGSTYR | 1.33 | 1.04E-02 | S387 |
| CL23073.Contig6_All | Aluminum induced protein with YGL and LRDR motifs | AT4G27450 | QVAHAPQELN**S**(ph)PR | 1.35 | 4.74E-02 | S101 |
| CL1277.Contig12_All | Linoleate 9S-lipoxygenase 5 | AT3G22400 | YDLYNDLGDPDNK**S**(ph)PRPTLGGPDSPYPYPR | 1.44 | 1.76E-03 | S51 |
| Unigene34796_All | 23.6 kDa heat shock protein, mitochondrial | AT4G25200 | EE**S**(ph)DDDSRR | 1.82 | 1.34E-03 | S58 |
| CL14785.Contig1_All | Heat shock 70 kDa protein 4 | AT3G12580 | LMPEPTAVALLYAQQQQQLLHDNMG**S**(ph)GIEK | 1.39 | 2.38E-02 | S302 |
| CL438.Contig2_All | Reticulon-like protein B1 | AT4G23630 | FHGSSSSSSD**S**(ph)DGEGK | 1.32 | 1.88E-03 | S54 |
| CL3317.Contig1_All | Serine/arginine-rich splicing factor SR45 | AT1G16610 | RH**S**(ph)R**S**(ph)PPPR | 1.99 | 1.73E-03 | S327; S329 |
| Unigene42224_All | Serine/arginine-rich splicing factor RSZ22 | AT4G31580 | GR**S**(ph)I**S**(ph)R**S**(ph)PVR | 1.33 | 1.78E-02 | S161; S163; S165 |
| CL5517.Contig12_All | Serine/arginine-rich SC35-like splicing factor SCL30 | AT3G55460 | GGGQHDGKR**S**(ph)PPLD**S**(ph)DG**S**(ph)PPR | 1.60 | 1.99E-03 | S253; S258; S261 |
| Unigene42056_All | Serine/arginine-rich SC35-like splicing factor SCL33 | AT1G55310 | RYSR**S**(ph)PAHR | 1.82 | 1.19E-03 | S205; S/Y |
| CL6723.Contig3_All | Splicing factor U2af small subunit A | AT1G27650 | SR**S**(ph)P**S**(ph)PQHR | 1.79 | 4.99E-04 | S198; S200 |
| CL6723.Contig3_All | Splicing factor U2af small subunit A | AT1G27650 | **S**(ph)PVREN**S**(ph)EER | 1.45 | 1.26E-03 | S298; S304 |
| CL6723.Contig3_All | Splicing factor U2af small subunit A | AT1G27650 | AR**S**(ph)PVREN**S**(ph)EER | 1.30 | 4.94E-02 | S316; S322 |
| Unigene37199_All | Serine/arginine-rich splicing factor RS2Z33 | AT2G37340 | AD**S**(ph)R**S**(ph)PADR | 1.35 | 1.73E-02 | S276; S278 |
| CL6216.Contig8_All | Pre-mRNA-splicing factor RDM16 | AT1G28060 | DRS**S**(ph)**S**(ph)RAPR | 1.31 | 1.81E-03 | S123; S124 |
| Unigene37195_All | Serine/arginine-rich splicing factor RS2Z33 | AT2G37340 | AD**S**(ph)R**S**(ph)PVDRER | 1.56 | 7.51E-02 | S258; S260 |
| CL3918.Contig2_All | DEAD-box ATP-dependent RNA helicase 40 | AT3G06480 | GR**S**(ph)Y**S**(ph)R**S**(ph)PDR | 1.43 | 5.61E-02 | S1188; S1190; S1192 |
| CL6723.Contig8_All | Splicing factor U2af small subunit B | AT5G42820 | GR**S**(ph)PVREN**S**(ph)EER | 1.32 | 4.83E-03 | S298; S304 |
| CL6723.Contig3_All | Splicing factor U2af small subunit A | AT1G27650 | GR**S**(ph)R**S**(ph)P**S**(ph)PQHR | 1.38 | 9.57E-03 | S248; S250; S252 |
| Unigene9165_All | High mobility group B protein 2 | AT1G20693 | AAPAAKDEDDDEEESDK**S**(ph)K | 1.31 | 2.36E-02 | S209 |
| CL7594.Contig7_All | RNA-binding (RRM/RBD/RNP motifs) family protein | AT5G55670 | **S**(ph)R**S**(ph)REVDHSK | 1.50 | 1.19E-03 | S251; S253 |
| Unigene6510_All | DNA topoisomerase 1 beta | AT5G55310 | RPHDGR**S**(ph)DD**S**(ph)DDDNRPLASR | 1.41 | 1.88E-03 | S121; S124 |
| CL5702.Contig5_All | HMG-Y-related protein A | AT1G14900 | AKG**S**(ph)PAGK**S**(ph)PAPAPK | 1.36 | 5.61E-03 | S127; S132 |
| Unigene24129_All | Kinase superfamily with octicosapeptide/ Phox/ Bem1p domain-containing protein | AT5G57610 | VKQH**T**(ph)LV**S**(ph)GGVR | 1.34 | 7.49E-03 | T996; S999 |
| CL6274.Contig2_All | FCS-Like Zinc finger 14 | AT5G20700 | RN**S**(ph)ADYSK | 1.43 | 1.23E-03 | S162 |
| CL9184.Contig2_All | Magnesium transporter MRS2-11, chloroplastic | AT5G22830 | AVAKEEEVEED**S**(ph)EEEER | 1.64 | 3.13E-03 | S107 |
| CL3378.Contig7_All | Probable aquaporin NIP4-2 | AT5G37820 | RLQ**S**(ph)QR**S**(ph)AADDFDHV | 1.31 | 1.40E-02 | S286; S289 |
| CL90.Contig2_All | AT3G56720 protein (Pre-mRNA-splicing factor) | AT3G56720 | GR**S**(ph)H**S**(ph)PVELKDDR | 1.39 | 2.87E-03 | S8; S10 |
| Unigene51511_All | NPL4-like protein 2 | AT2G47970 | ALAPAG**S**(ph)FGK | 1.31 | 1.44E-03 | S244 |
| CL8070.Contig2_All | Pre-mRNA-splicing factor | AT3G49601 | RHD**S**(ph)D**S**(ph)DGYAHK | 1.31 | 1.02E-02 | S454; S456 |
| CL12186.Contig3_All | Arginine/glutamate-rich 1 protein | AT5G13340 | DR**S**(ph)R**S**(ph)PGPSR | 1.31 | 6.05E-03 | S72; S74 |
| CL7713.Contig3_All | LOW protein: zinc finger CCCH domain protein | AT5G53440 | DR**S**(ph)D**S**(ph)DEDPRPR | 1.34 | 8.76E-02 | S20; S22 |
| CL12226.Contig5_All | RNA recognition motif XS domain protein | AT3G22430 | RS**S**(ph)R**S**(ph)P**S**(ph)PAYR | 1.32 | 1.78E-02 | S69; S71; S73 |
| CL7590.Contig2_All | Abscisic acid receptor PYL5 | AT5G05440 | **S**(ph)R**S**(ph)PPRPSLPPPPPLGLSRPER | 1.38 | 1.77E-02 | S73; S75 |
| Unigene29452_All | Tetratricopeptide repeat (TPR)-like superfamily protein | AT1G01320 | E**S**(ph)QSEHESDNTGKDDLPDK | 1.32 | 6.94E-03 | S1153 |
| Down-regulated phosphopeptides | | | | | | |
| Unigene24022_All | Ribulose bisphosphate carboxylase large chain | ATCG00490 | AIKFEFEPVD**T**(ph)IDN | 0.70 | 1.73E-03 | T474 |
| CL5834.Contig3_All | Sucrose phosphate synthase 4F | AT4G10120 | IN**S**(ph)EVRLV**S**(ph)DDEEEQTK | 0.54 | 1.47E-03 | S176; S182 |
| CL19104.Contig4_All | Phosphoenolpyruvate carboxykinase | AT4G37870 | **S**(ph)AP**T**(ph)TPIK | 0.75 | 1.41E-03 | S87; T90 |
| CL840.Contig1_All | Oleosin-B3-like protein | AT1G13930 | QY**S**(ph)**S**(ph)GGAEKEK | 0.50 | 1.14E-03 | S76; S77 |
| CL14676.Contig4_All | Dehydrin HIRD11 | AT1G54410 | ITGEHGDK**S**(ph)**S**(ph)DDHKEK | 0.72 | 7.45E-03 | S64; S65 |
| CL5517.Contig7_All | Serine/arginine-rich SC35-like splicing factor SCL30 | AT3G55460 | SPAPDSDG**S**(ph)PSR | 0.71 | 5.18E-02 | S350 |
| CL6783.Contig3_All | Serine/arginine-rich splicing factor RS41 | AT5G52040 | SGGDDE**Y**(ph)SGGGYK | 0.73 | 3.20E-03 | S/Y |
| CL6783.Contig5_All | Serine/arginine-rich splicing factor RS31A | AT2G46610 | SGTDDYDR**S**(ph)EGR | 0.66 | 1.44E-03 | S239 |
| CL15459.Contig2_All | Splicing factor PWI domain-containing protein | AT1G60200 | TSNETEKAETG**S**(ph)PDR | 0.75 | 3.39E-03 | S292 |
| CL4905.Contig3_All | SAP domain-containing protein | AT4G39680 | RLFESIQSEEA**S**(ph)DESPEDAGDNEAVEEGAK | 0.76 | 3.22E-03 | S173 |
| CL2386.Contig1_All | 40S ribosomal protein S6-2 | AT5G10360 | DRR**S**(ph)E**S**(ph)LAK | 0.76 | 2.37E-03 | S229; S231 |
| CL14197.Contig4_All | Nucleolar GTP-binding protein 1 | AT1G50920 | GSRS**S**(ph)G**S**(ph)HEDTEK | 0.75 | 6.78E-02 | S1145; S1147 |
| CL12618.Contig5_All | Root phototropism protein 2 | AT2G30520 | SAGAGIGHDDDDEAR**S**(ph)EAGSAR | 0.59 | 4.83E-03 | S479 |
| CL12618.Contig5_All | Root phototropism protein 2 | AT2G30520 | SAGAGIGHDDDDEAR**S**(ph)EAG**S**(ph)AR | 0.49 | 3.35E-02 | S479; S483 |
| CL3553.Contig3_All | Calmodulin-binding protein 60 B | AT5G57580 | IQGSENR**S**(ph)SPK | 0.75 | 1.81E-03 | S159 |
| CL6783.Contig3_All | Putative glycine-rich cell wall protein | AT4G36230 | **S**(ph)GDDYDNK | 0.65 | 1.15E-03 | S7 |
| Unigene42348_All | Chaperone DnaJ-domain superfamily protein | AT4G36520 | IF**S**(ph)PE**S**(ph)SPK | 0.64 | 3.24E-03 | S419; S422 |

Note: ND-H, non-drought priming + subsequent heat stress; D-H, drought priming + subsequent heat stress. Locus number are the correspondent Arabidopsis IDs blasted against The Arabidopsis Information Resource (TAIR) using sequences of identified UniProt IDs.

Table S4 Details of the phosphorylation motifs identified within differently regulated phosphoproteins under drought priming and heat stress treatments

| Treatment comparison | Motif | Score | Fold enrichment | FG | BG | Adjusted p-value |
| --- | --- | --- | --- | --- | --- | --- |
| D-VS ND- Up-regulated | ……GS…… | 7.52 | 10.0 | 10/32 | 1/32 | 7.4e-2 |
| D-H VS D-C Up-regulated | ……DSD…… | 20.58 | 17.0 | 17/95 | 1/95 | 5.6e-3 |
| D-H VS D-C Up-regulated | ……S..E…… | 7.77 | 3.6 | 24/78 | 8/94 | 1.9e-2 |
| D-H VS D-C Up-regulated | ……SP… | 8.47 | 4.6 | 20/54 | 7/86 | 2.2e-3 |
| D-H VS D-C Down-regulated | ……S..E…… | 6.71 | 6.0 | 12/29 | 2/29 | 6.0e-2 |
| D-H VS ND-C Up-regulated | ……SP… | 13.20 | 6.3 | 25/77 | 4/77 | 1.0e-3 |
| D-H VS ND-C Up-regulated | ……S..E…… | 9.76 | 4.2 | 24/52 | 8/73 | 7.4e-4 |
| ND-H VS D-C Up-regulated | ……SP…… | 9.60 | 5.3 | 21/77 | 4/77 | 1.6e-2 |
| ND-H VS D-C Up-regulated | ……SD…… | 11.44 | 3.7 | 31/56 | 11/73 | 1.1e-4 |
| ND-H VS ND-C Up-regulated | ……SP…… | 13.55 | 6.3 | 25/64 | 4/64 | 5.7e-4 |
| ND-H VS ND-C Up-regulated | ……SD…… | 14.42 | 8.1 | 17/39 | 4/60 | 8.9e-6 |

Note: Graphic presentations of phosphorylation motifs were shown in Fig. 3.
